# Gam-COVID-Vac (Sputnik V) and Pfizer-BioNTech Vaccines Adverse Events following Immunization in Patients Affected by Parkinson’s Disease and Multiple Sclerosis: A Longitudinal Study

**DOI:** 10.3390/vaccines10030370

**Published:** 2022-02-26

**Authors:** Giorgia Soldà, Edoardo Barvas, Jacopo Lenzi, Zeno Di Valerio, Giusy La Fauci, Susanna Guttmann, Rossano Riccardi, Maria Pia Fantini, Aurelia Salussolia, Marco Montalti, Davide Gori

**Affiliations:** 1 School of Hygiene and Preventive Medicine, Department of Biomedical and Neuromotor Sciences, Public Health and Medical Statistics, University of Bologna, 40126 Bologna, Italy; giorgia.solda@studio.unibo.it (G.S.); zeno.divalerio@studio.unibo.it (Z.D.V.); aurelia.salussolia@studio.unibo.it (A.S.); marco.montalti7@studio.unibo.it (M.M.); 2 San Marino Neurological Unit, State Hospital of the Republic of San Marino, 47893 Cailungo, San Marino; edoardo.barvas@iss.sm (E.B.); susanna.guttmann@iss.sm (S.G.); 3 Unit of Hygiene, Department of Biomedical and Neuromotor Sciences, Public Health and Medical Statistics, University of Bologna, 40126 Bologna, Italy; jacopo.lenzi2@unibo.it (J.L.); mariapia.fantini@unibo.it (M.P.F.); davide.gori4@unibo.it (D.G.); 4 San Marino Central Pharmacy, San Marino Hospital, 47893 Cailungo, San Marino; rossano.riccardi@iss.sm

**Keywords:** AEFI, adverse event, vaccination, Gam-COVID-Vac, Pfizer-BioNTech, Parkinson’s disease, Multiple Sclerosis

## Abstract

The Republic of San Marino COVID-19 vaccination campaign used Gam-COVID-Vac and Pfizer-BioNTech vaccines. To assess adverse events following immunization (AEFIs), approximately 6000 vaccine recipients were monitored by the ROCCA study, including subgroups with Parkinson’s Disease (PD) and Multiple Sclerosis (MS). The purpose of this study is to evaluate short-term AEFIs through a 1-month follow-up. We conducted a longitudinal study, using active surveillance to evaluate the safety profiles of COVID-19 vaccines in PD and MS patients. Participants were actively administered a standardized online questionnaire to collect information on AEFIs. Among all PD and MS assisted by the San Marino hospital, a total of 82 patients completed the questionnaires. One week after administration of the first dose, vaccine recipients reported AEFIs in 26% of cases in the PD group, 67% in the MS group, and 68% in the control group. Participants reported slightly higher rates of AEFIs after dose 2 compared with dose 1, being 29%, 75%, and 78% for PD, MS, control group, respectively. Most of the reported symptoms were mild. Patients with PD and MS reported few AEFIs after administration of the COVID-19 vaccines. The frequency of AEFIs in the PD population was significantly lower than in the control group.

## 1. Introduction

The Republic of San Marino COVID-19 vaccination campaign started on 25 February 2021, involving, in the first phase, health care workers and most vulnerable populations, with a total of 68,749 doses given by 10 February 2022 [1,2].

The Gam-COVID-Vac vaccine was the most used vaccine in the Republic of San Marino’s vaccination campaign, followed by the Pfizer-BioNTech vaccine; by December 2021, 83.4% of the eligible population completed the immunization cycle [2].

Thanks to the ROCCA active vaccine surveillance study program, around 6000 recipients were monitored for adverse events following immunization (AEFIs), including subgroups presenting neurological diseases [3].

Persons with chronic or progressive neurologic diseases such as Parkinson’s disease (PD) and Multiple Sclerosis (MS) face significant declines in mobility and cognition, resulting in a loss of independence and compromised health-related quality of life over the course of the disease [4,5].

The risk of SARS-CoV-2 infection causing serious, life-threatening disease seems higher for Parkinson’s Disease (PD) patients [6]. Indeed, although the risk of becoming infected is not higher than in the general population, the risk of dying in conjunction with SARS-CoV-2 infection seems to be higher [7]; in particular, the frailty caused by advanced PD poses an increased risk of mortality with a significant effect of co-occurrence of dementia, hypertension, and disease duration [8].

Moreover, motor and non-motor symptoms can worsen significantly as a consequence of SARS-CoV-2 infection, possibly due to infection-related mechanisms and impaired pharmacokinetics of dopaminergic therapy [9]. Both viral vector and mRNA-based COVID-19 vaccines are not known or expected to interact with the neurodegenerative process in PD or to interfere with the current therapies of PD and are therefore recommended to people with PD [10,11]. However, to the best of our knowledge, there is currently not enough scientific data specifically regarding the safety of COVID-19 vaccinations for people with PD, and a study of the safety profile of the COVID-19 vaccines in this population is required.

For what concerns Multiple Sclerosis (MS), patients affected by it seem to have similar incidence, risk factors, and outcomes for COVID-19 as the general population [12]. A previous study on the safety of the Pfizer-BioNTech vaccine in MS indicates a safety profile of the vaccine in people with MS similar to that reported in the general population [13].

Preliminary results on the Republic of San Marino general population aged ≥ 60 years were published and showed a high tolerability profile in terms of short-term AEFIs [3]. However, there has not been a specific focus on subgroups with comorbidities, especially neurological ones, yet [14].

The aim of this study is to assess potential short-term AEFIs of Gam-COVID-Vac and Pfizer-BioNTech vaccines through a 1-month follow-up of the recipients affected by PD and MS, confronting them with short-term AEFIs of a control group of the population from the main ROCCA active surveillance study.

## 2. Materials and Methods

### 2.1. Study Design and Participants

We conducted a longitudinal study in the Republic of San Marino, using active surveillance to evaluate Gam-COVID-Vac (The Russian Direct Investment Fund, Moscow, Russia) and Pfizer-BioNTech vaccines safety profiles in PD and MS groups compared to a non-neurological one. Gam-COVID-Vac vaccine consists of two doses, respectively 0.5 mL of rAd26 and 0.5 mL of rAd5 [15], while Pfizer-BioNTech (Pfizer, New York City, NY, USA) has two 30-μg doses of BNT162b2 [16]. Both are administered intramuscularly 21 days apart.

The recruitment of vaccine recipients was performed by the neurological team of the San Marino State Hospital, who contacted all patients affected by PD and MS assisted by the Neurological Department, and on-site by physicians immediately after the vaccine administration for the control group. All participants provided informed consent for study participation. Eligibility criteria were defined as age over 18, having had at least one dose of Gam-COVID-Vac or Pfizer-BioNTech vaccine administered, being covered by the national health insurance. For those patients diagnosed with Parkinson’s Disease Dementia (PD-D), the questionnaire was administered to the patients’ caregivers.

To assess AEFIs, we compared the responses of patients with neurological disorders (PD or MS) with those of a random control group of non-neurological patients matched with a 1:1 ratio according to sex, age, concomitant medical conditions, and food or drug allergies from the population of the main ROCCA active surveillance study.

The study protocol was approved by the Ethics Committee for Research and Experimentation of the Republic of San Marino under approval number 30/CERS/2021 on 17 March 2021. This study follows the criteria of Strengthening the Reporting of Observational Studies in Epidemiology (STROBE) [17].

### 2.2. Outcomes

The main outcome was to describe in real-world context the safety profile of the Gam-COVID-Vac and Pfizer-BioNTech vaccines in PD and MS affected patients, based on the number of participants reporting AEFIs and the severity of AEFIs, comparing them with a non-neurological recipients’ group.

### 2.3. Procedure and Questionnaire

Participants were actively administered a standardized online questionnaire to collect information on potential post-vaccine AEFIs.

Vaccine recipients were asked to complete the questionnaire at regular intervals, one week (Q1) and one month (Q2) after the injection. This allowed the collection of near real-time, short-term patient-reported safety data.

Q1 collected demographic information, patient code, anamnestic data, date of the first injection, vaccine brand, the potential AEFIs occurring in the week after the first dose, and the severity/impact of the symptoms (including the need for medical assistance and hospitalization). Q2 addressed the aforementioned questions one week after the second dose. Patient codes were also provided at Q2 so that the questionnaires could be clearly linked to each other. To minimize missing data, answers to the relevant variables were mandatory in both questionnaires.

Most of the AEFI lists and questions were derived from the European Medicines Agency (EMA) funded active pharmacovigilance project Covid Vaccine Monitor [18]. The events listed are similar to typical AEFIs for vaccines identified as potential by the competent regulatory authorities. The clinical characteristics, frequency, and severity of all adverse events were reported using a questionnaire based on the Common Criteria for Terminology for Adverse Events (CTCAE) version 5–0 [19]. Grade 1 indicates the presence of mild events that do not require intervention, Grade 2 that events are moderate, Grade 3 that events are severe or medically significant, and Grade 4 that events require emergency intervention. Grade 5 (death) was not directly addressed in the questionnaire due to its nature.

### 2.4. Data Collection

The questionnaire collection lasted from 4 March 2021 to 10 August 2021. Data collection was carried out through face-to-face or telephone interviews or online access to the e-questionnaire by a link sent via e-mail.

### 2.5. Medical History Assessment

The collection through the e-questionnaire of the patient code allowed the verification of the personal data, medical history, and drug therapy of each participant. The patient code was also used to verify access to the national health service after vaccination.

### 2.6. Statistical Analysis

Numerical and categorical variables were summarized as mean ± standard deviation and as count (percentage), respectively. Differences in AEFI occurrence between the three study groups (PD, MS and controls) were evaluated with logistic regression analysis. Regression estimates were obtained using the conditional maximum likelihood method, which is a small-sample alternative to the standard maximum-likelihood-based logistic regression estimator. The likelihood was calculated relative to each matched pair, that is, a conditional likelihood was used [20]. All analyses were carried out using Stata software, version 15 [21]. The significance level was set at 0.05, and all tests were two-sided.

## 3. Results

### 3.1. Participants

We collected a total of 57 responses from PD patients and 103 from MS patients, all outpatients from the Department of Neurology of the State Hospital of the Republic of San Marino were eligible for inclusion in the study considering our criteria. Of these, a total of 82 patients with neurological disorders completed Q1 and Q2, including 34 (60%) subjects with PD and 48 (47%) subjects with MS. In the overall sample, 61 (74%) subjects received the Gam-COVID-Vac vaccine and 21 (26%) subjects received Pfizer-BioNTech vaccine. The control group consisted of 82 non-neurological patients paired with a 1:1 ratio according to sex, 10-year age group, number of concomitant conditions (0, 1, ≥2), and food or drug allergies. In the control group, all 82 (100%) individuals received the Gam-COVID-Vac vaccine.

### 3.2. Baseline Characteristics

The sociodemographic and clinical characteristics of the 164 patients included in the study—including the control group—are presented in Table 1. The neurological patients’ sample was analyzed separately according to their neurological disorder. In the group of subjects with PD, a total of 15 (44%) were female and the mean age was 73 ± 9 years; in the group of subjects with MS, 40 (83%) were female and the mean age was 54 ± 14 years. In the control group, 55 (67%) were female and the mean age was 62 ± 16.

In the group of patients with PD, more than half of the participants (68%) had at least one underlying medical condition besides PD; in particular, cardiovascular disorders were the most frequent coexisting conditions. Similarly, in the group of patients with MS, more than half of the participants (75%) had at least one underlying medical condition besides MS, but in this group immunosuppression was the most frequent coexisting condition (54%). Individuals with drug or food allergies were nine (26%) in the first group, six (13%) in the second group, and 15 (18%) in the control group.

Previous SARS-CoV-2 infection was reported by 3–4% of participants. On the day of their first Gam-COVID-Vac or Pfizer-BioNTech vaccination, all PD patients and 83% of MS patients reported taking regular medical therapy.

### 3.3. AEFI after the First Dose

One week after administration of the first dose, vaccine recipients described both local and systemic events in 26% of cases in the PD group, 67% in the MS group, and 68% in the control group.

In the group with PD, 15% reported local AEFIs, and 21% reported systemic ones. Among the local reported events, the more frequent were pain at the injection site (15%) and nodules at the injection site (3%). The most frequently reported systemic AEFIs were fatigue (9%), joint pain (6%), malaise (6%), and headache (6%). All reported symptoms, both local and systemic, were Grade 1.

In the MS patient group, 40% described local events, and 46% of patients described systemic events. The most frequent local AEFIs were pain (37%), nodules (4%), itching (4%), and warmth (2%). Almost all local symptoms were Grade 1, except for 6% of cases reporting Grade 2 and Grade 4. The most frequently reported systemic symptoms were fatigue (33%), chills (25%), joint pain (17%), muscle pain (15%), malaise (12%), and headache (10%). Among patients who reported systemic symptoms, 17% reported Grade 2, 2% Grade 4, and all others Grade 1. For all other AEFIs see Table 2.

Lastly, in the control group 56 (68%) individuals reported local or/and systemic symptoms. Among them, local AEFIs were reported in 43% of cases (42% pain, 4% swelling, 2% nodules), and systemic AEFIs in 49% (29% fatigue, 27% headache, 22% muscle pain, 21% joint pain, and other less frequent symptoms—see Table 2). Overall 56% of the symptoms described were Grade 1, 11% Grade 2, and 1% Grade 3.

### 3.4. AEFI after the Second Dose

All study participants reported slightly higher rates of local or systemic AEFIs after dose 2 compared with dose 1. Symptoms were reported in 10 (29%) patients with PD, in 36 (75%) patients with MS, and 64 (78%) subjects in the control group. All data collected on AEFIs after the second dose in the two groups of our study compared with those in the control group are shown in Table 3.

Specifically, only 12% of the subjects with PD reported local symptoms, including pain (9%), and nodules (3%) at the injection site. Meanwhile, systemic symptoms were reported by 21% of patients with PD, most frequently malaise (12%), fatigue (9%), and joint pain (6%). Grading of all AEFIs was always graded 1, as after the first dose.

Instead, in the group of patients with MS 30 (62%) patients reported local AEFIs, of which were mainly pain (58%), nodules (6%), warmth (4%), and itching (4%) at the injection site. In 58% of cases, local symptoms were Grade 1, and in 4% Grade 2. Systemic AEFIs were described in 46% of MS patients, the most reported were fatigue (31%), malaise (27%), chills (25%), headache (23%), joint and muscle pain (21%), and fever (17%). Among these, 27% of patients reported that systemic symptoms were at most Grade 1, 17% Grade 2, and 2% Grade 3.

In the control group, both local and systemic symptoms were reported by 64 (77%) individuals. Subjects described local AEFIs in 62% of cases, including 57% pain, 15% nodules, and 8% itching. Systemic AEFIs were reported in 57% of individuals (mainly 41% malaise, 28% muscle pain, 26% fatigue, 25% joint pain). Most symptoms were Grade 1 or 2. For the complete list of AEFIs, see Table 3.

### 3.5. Comparison between Different Neurological Diseases

As shown in Table 4, there was no significantly higher or lower risk of any AEFI for both PD- and MS-affected recipients after the first dose (OR 0.40 PD and 0.41 MS).

However, the frequency of any AEFI and local AEFIs was significantly lower in patients with Parkinson’s disease after the second dose. In these patients, the odds ratio was 0.19 (0.03–0.66) for any AEFI, and 0.12 (0.01–0.51) for the frequency of local AEFIs (*p* < 0.05).

For what concerns MS-affected patients, the odds ratio of any AEFI after the second dose was 0.73, but this result was not statistically significant.

## 4. Discussion

All vaccine recipients affected by PD and MS were contacted by the neurological team, and eventually, the study included 60% of all patients with PD assisted by the San Marino State Hospital and 48% of those with MS.

Overall, even though our sample size was quite small, our findings showed that PD and MS diagnosed participants reported fewer or similar AEFIs compared to the control group, and almost all of them were graded 1 or 2 in intensity and/or duration. This was even more evident in the PD group.

For both doses in both the PD and MS groups, recipients reported fewer AEFIs than in the control group, with the only exception being patients with MS reporting higher rates of systemic events after the 1st dose. However, the only statistically significant results concern the PD sample and the AEFIs reported after the 2nd dose.

From these results, it would seem that PD is associated with a lower rate of AEFIs, especially after the second dose (OR = 0.12). So far, the approved mRNA-based vaccines and the viral vector vaccines under development induce immunization through mechanisms that are not known to interact with the neurodegenerative process in PD [11].

However, the size of our samples is not particularly big and this certainly represents one of the limitations of the study. This limitation underlines the need to conduct larger-scale studies on patients with comorbidities. In addition, the very small number of Pfizer-BioNTech recipients in our study—six in the PD group, 15 in the MS group, and none in the control group)—prevents us from performing a robust statistical analysis to compare the safety profiles of the two vaccines. Moreover, although the inclusion criteria were met for all participants, the presence of neurocognitive disorders could have influenced the reliability of the questionnaires, especially for PD patients [22]. In fact, among patients with PD, cognitive deficits involving attention, executive functions, memory, and language are frequently observed [23], with an average prevalence of dementia ranging from 26% to 78.2% [24] in longitudinal studies. Moreover, among PD patients without dementia, mild cognitive impairment (MCI) is common in the early stages, with a prevalence of 25% [25].

In our study, for patients diagnosed with neurocognitive disorders due to PD or MS, the questionnaire was administered to the patients’ caregivers. However, the presence of neurocognitive disorders might still have influenced patients’ ability to report adverse events to the caregiver, and therefore the reliability of the questionnaires.

## 5. Conclusions

Our data showed few AEFIs reported after the administration of Gam-COV-Vac and Pfizer-BioNTech vaccines in patients with Parkinson’s disease and Multiple Sclerosis. Additionality, the frequency of AEFIs in the population with Parkinson’s disease was significantly lower than in the control group.

## Figures and Tables

**Table 1:** Characteristics of neurological and 82 non-neurological patients paired in a 1:1 ratio according to sex, 10-year age group, number of concomitant conditions (0, 1, ≥2), and food or drug allergies. Values are counts (percentages) or mean ± standard deviation.

	Parkinson’s Disease	Multiple Sclerosis	Control
	(*n* = 34)	(*n* = 48)	(*n* = 82)
Vaccine			
Gam-COVID-Vac	28 (82%)	33 (69%)	82 (100%)
Pfizer-BioNTech	6 (18%)	15 (31%)	0 (0%)
Female sex	15 (44%)	40 (83%)	55 (67%)
Age, years	73 ± 9	54 ± 14	62 ± 16
Comorbidities, number			
0	11 (32%)	12 (25%)	23 (28%)
1	7 (21%)	13 (27%)	20 (24%)
≥2	16 (47%)	23 (48%)	39 (48%)
Comorbidities			
Hypertension	13 (38%)	9 (19%)	31 (38%)
Obesity (BMI ≥30 kg/m^2^)	5 (15%)	8 (17%)	18 (22%)
Cardiovascular diseases	6 (18%)	3 (6%)	21 (26%)
Immunosuppression	1 (3%)	26 (54%)	1 (1%)
Osteoarticular diseases	3 (9%)	5 (10%)	11 (13%)
Diabetes mellitus	6 (18%)	3 (6%)	7 (9%)
Malignant tumor	3 (9%)	0 (0%)	5 (6%)
Respiratory diseases	2 (6%)	1 (2%)	3 (4%)
Mental disorders	0 (0%)	1 (2%)	4 (5%)
Nephropathy	1 (3%)	2 (4%)	1 (1%)
Neurological diseases (excluding PD and MS)	0 (0%)	0 (0%)	3 (4%)
Liver diseases	0 (0%)	2 (4%)	0 (0%)
Other conditions	7 (21%)	12 (25%)	27 (33%)
Ongoing drug therapies	34 (100%)	40 (83%)	56 (68%)
Food or drug allergies	9 (26%)	6 (13%)	15 (18%)
Previous SARS-CoV-2 infection	1 (3%)	2 (4%)	3 (4%)

PD; Parkinson’s Disease; MS, Multiple Sclerosis; SARS-CoV-2, severe acute respiratory syndrome coronavirus 2.

**Table 2:** AEFIs after the first dose in neurological patients and in 82 non-neurological patients paired in a 1:1 ratio according to sex, 10-year age group, number of concomitant conditions (0, 1, ≥2), and food or drug allergies.

	Parkinson’s Disease	Multiple Sclerosis	Control
	(*n* = 34)	(*n* = 48)	(*n* = 82)
Highest grade, any AEFIs			
No reactions	25 (74%)	16 (33%)	26 (32%)
Grade I	9 (26%)	27 (56%)	46 (56%)
Grade II	0 (0%)	3 (6%)	9 (11%)
Grade III	0 (0%)	0 (0%)	1 (1%)
Grade IV	0 (0%)	2 (4%)	0 (0%)
Highest grade, local AEFIs			
No reactions	29 (85%)	29 (60%)	47 (57%)
Grade I	5 (15%)	15 (31%)	32 (39%)
Grade II	0 (0%)	2 (4%)	3 (4%)
Grade IV	0 (0%)	2 (4%)	0 (0%)
Local pain			
No	29 (85%)	30 (63%)	48 (59%)
Grade I	5 (15%)	14 (29%)	31 (38%)
Grade II	0 (0%)	3 (6%)	3 (4%)
Grade IV	0 (0%)	1 (2%)	0 (0%)
Local redness			
No	34 (100%)	48 (100%)	82 (100%)
Local warmth			
No	34 (100%)	47 (98%)	82 (100%)
Grade I	0 (0%)	1 (2%)	0 (0%)
Local swelling			
No	34 (100%)	47 (98%)	79 (96%)
Grade I	0 (0%)	1 (2%)	3 (4%)
Local itching			
No	34 (100%)	46 (96%)	82 (100%)
Grade I	0 (0%)	1 (2%)	0 (0%)
Grade IV	0 (0%)	1 (2%)	0 (0%)
Nodules			
No	33 (97%)	46 (96%)	80 (98%)
Grade I	1 (3%)	1 (2%)	2 (2%)
Grade II	0 (0%)	1 (2%)	0 (0%)
Local ecchymosis			
No	34 (100%)	47 (98%)	82 (100%)
Grade I	0 (0%)	1 (2%)	0 (0%)
Highest grade, systemic AEFIs			
No reactions	27 (79%)	26 (54%)	42 (51%)
Grade I	7 (21%)	13 (27%)	34 (41%)
Grade II	0 (0%)	8 (17%)	5 (6%)
Grade III	0 (0%)	0 (0%)	1 (1%)
Grade IV	0 (0%)	1 (2%)	0 (0%)
Fever			
No	34 (100%)	44 (92%)	72 (88%)
Grade I	0 (0%)	2 (4%)	9 (11%)
Grade II	0 (0%)	1 (2%)	1 (1%)
Grade III	0 (0%)	1 (2%)	0 (0%)
Chills			
No	34 (100%)	36 (75%)	70 (85%)
Grade I	0 (0%)	8 (17%)	10 (12%)
Grade II	0 (0%)	3 (6%)	2 (2%)
Grade IV	0 (0%)	1 (2%)	0 (0%)
Joint pain			
No	32 (94%)	40 (83%)	65 (79%)
Grade I	2 (6%)	8 (17%)	14 (17%)
Grade II	0 (0%)	0 (0%)	3 (4%)
Muscle pain			
No	34 (100%)	41 (85%)	64 (78%)
Grade I	0 (0%)	6 (13%)	14 (17%)
Grade II	0 (0%)	0 (0%)	4 (5%)
Grade III	0 (0%)	1 (2%)	0 (0%)
Malaise			
No	32 (94%)	42 (88%)	70 (85%)
Grade I	2 (6%)	4 (8%)	10 (12%)
Grade II	0 (0%)	2 (4%)	2 (2%)
Fatigue			
No	31 (91%)	32 (67%)	58 (71%)
Grade I	3 (9%)	10 (21%)	20 (24%)
Grade II	0 (0%)	5 (10%)	3 (4%)
Grade III	0 (0%)	1 (2%)	1 (1%)
Headache			
No	32 (94%)	43 (90%)	60 (73%)
Grade I	2 (6%)	5 (10%)	21 (26%)
Grade II	0 (0%)	0 (0%)	1 (1%)
Nausea and/or vomiting			
No	34 (100%)	45 (94%)	74 (90%)
Grade I	0 (0%)	2 (4%)	7 (9%)
Grade II	0 (0%)	0 (0%)	1 (1%)
Grade III	0 (0%)	1 (2%)	0 (0%)
Other AEFIs			
No	34 (100%)	45 (94%)	76 (93%)
Grade I	0 (0%)	1 (2%)	4 (5%)
Grade II	0 (0%)	1 (2%)	2 (2%)
Grade IV	0 (0%)	1 (2%)	0 (0%)

**Table 3:** AEFIs after the second dose in neurological patients and in 82 non-neurological patients paired in a 1:1 ratio according to sex, 10-year age group, number of concomitant conditions (0, 1, ≥2), and food or drug allergies.

	Parkinson’s Disease	Multiple Sclerosis	Control
	(*n* = 34)	(*n* = 48)	(*n* = 82)
Highest grade, any AEFIs			
No reactions	24 (71%)	12 (25%)	18 (22%)
Grade I	10 (29%)	26 (54%)	52 (63%)
Grade II	0 (0%)	9 (19%)	9 (11%)
Grade III	0 (0%)	1 (2%)	2 (2%)
Grade IV	0 (0%)	0 (0%)	1 (1%)
Highest grade, local AEFIs			
No reactions	30 (88%)	18 (38%)	31 (38%)
Grade I	4 (12%)	28 (58%)	45 (55%)
Grade II	0 (0%)	2 (4%)	6 (7%)
Local pain			
No	31 (91%)	20 (42%)	35 (43%)
Grade I	3 (9%)	26 (54%)	43 (52%)
Grade II	0 (0%)	2 (4%)	4 (5%)
Local redness			
No	34 (100%)	48 (100%)	78 (95%)
Grade I	0 (0%)	0 (0%)	3 (4%)
Grade II	0 (0%)	0 (0%)	1 (1%)
Local warmth			
No	34 (100%)	46 (96%)	77 (94%)
Grade I	0 (0%)	2 (4%)	4 (5%)
Grade II	0 (0%)	0 (0%)	1 (1%)
Local swelling			
No	34 (100%)	47 (98%)	76 (93%)
Grade I	0 (0%)	1 (2%)	5 (6%)
Grade II	0 (0%)	0 (0%)	1 (1%)
Local itching			
No	34 (100%)	46 (96%)	75 (91%)
Grade I	0 (0%)	2 (4%)	6 (7%)
Grade II	0 (0%)	0 (0%)	1 (1%)
Nodules			
No	33 (97%)	45 (94%)	70 (85%)
Grade I	1 (3%)	3 (6%)	12 (15%)
Local ecchymosis			
No	34 (100%)	47 (98%)	81 (99%)
Grade I	0 (0%)	1 (2%)	1 (1%)
Highest grade, systemic AEFIs			
No reactions	27 (79%)	26 (54%)	35 (43%)
Grade I	7 (21%)	13 (27%)	38 (46%)
Grade II	0 (0%)	8 (17%)	7 (9%)
Grade III	0 (0%)	1 (2%)	1 (1%)
Grade IV	0 (0%)	0 (0%)	1 (1%)
Fever			
No	33 (97%)	40 (83%)	72 (88%)
Grade I	1 (3%)	6 (13%)	8 (10%)
Grade II	0 (0%)	1 (2%)	1 (1%)
Grade III	0 (0%)	1 (2%)	1 (1%)
Chills			
No	33 (97%)	36 (75%)	68 (83%)
Grade I	1 (3%)	10 (21%)	11 (13%)
Grade II	0 (0%)	1 (2%)	3 (4%)
Grade III	0 (0%)	1 (2%)	0 (0%)
Joint pain			
No	32 (94%)	38 (79%)	62 (76%)
Grade I	2 (6%)	7 (15%)	16 (20%)
Grade II	0 (0%)	2 (4%)	3 (4%)
Grade III	0 (0%)	1 (2%)	0 (0%)
Grade IV	0 (0%)	0 (0%)	1 (1%)
Muscle pain			
No	34 (100%)	38 (79%)	59 (72%)
Grade I	0 (0%)	6 (13%)	17 (21%)
Grade II	0 (0%)	3 (6%)	5 (6%)
Grade III	0 (0%)	1 (2%)	1 (1%)
Malaise			
No	30 (88%)	35 (73%)	48 (59%)
Grade I	4 (12%)	8 (17%)	29 (35%)
Grade II	0 (0%)	4 (8%)	5 (6%)
Grade III	0 (0%)	1 (2%)	0 (0%)
Fatigue			
No	31 (91%)	33 (69%)	61 (74%)
Grade I	3 (9%)	13 (27%)	18 (22%)
Grade II	0 (0%)	2 (4%)	3 (4%)
Headache			
No	33 (97%)	37 (77%)	63 (77%)
Grade I	1 (3%)	10 (21%)	17 (21%)
Grade II	0 (0%)	1 (2%)	2 (2%)
Nausea and/or vomiting			
No	33 (97%)	44 (92%)	71 (86%)
Grade I	1 (3%)	2 (4%)	8 (10%)
Grade II	0 (0%)	2 (4%)	3 (4%)
Other AEFIs			
No	33 (97%)	48 (100%)	77 (94%)
Grade I	1 (3%)	0 (0%)	3 (4%)
Grade II	0 (0%)	0 (0%)	1 (1%)
Grade III	0 (0%)	0 (0%)	1 (1%)

**Table 4:** Results of conditional exact logistic regression analysis: risk of first- and second-dose AEFIs associated with Parkinson’s Disease and Multiple Sclerosis diagnosis. All estimates controlled for pairing factors (sex, age, comorbidities, allergies) and type of vaccine received (Gam-COVID-Vac or Pfizer-BioNTech).

	Any AEFI	Systemic AEFIs	Local AEFIs
	OR (95% CI)	OR (95% CI)	OR (95% CI)
First dose			
Neurological diseases			
No	1.00	1.00	1.00
Parkinson’s Disease	0.40 (0.11–1.19)	0.62 (0.16–2.12)	0.36 (0.09–1.20)
Multiple Sclerosis	0.41 (0.08–1.62)	1.30 (0.43–4.09)	0.42 (0.13–1.17)
Second dose			
Neurological diseases			
No	1.00	1.00	1.00
Parkinson’s Disease	0.19 * (0.03–0.66)	0.42 (0.12–1.29)	0.12 * (0.01–0.51)
Multiple Sclerosis	0.73 (0.15–3.42)	0.66 (0.20–2.05)	0.95 (0.28–3.17)

Notes: OR, odds ratio; CI, confidence interval. * statistically significant (*p* < 0.05).

## Data Availability

Not applicable.

## Abbreviation

ROCCA: RSM Observatory for COVID vaccination Campaigns monitoring Adverse events.
